# Retraction: In Vivo Antioxidant and Antiulcer Activity of *Parkia speciosa* Ethanolic Leaf Extract against Ethanol-Induced Gastric Ulcer in Rats

**DOI:** 10.1371/journal.pone.0294012

**Published:** 2023-11-10

**Authors:** The PLOS ONE Editors

Following the publication of this article [1], concerns were raised regarding reuse of results presented in Figs 1, 3, 4, 5, and 6. Specifically,

- Fig 1, the following panels appear similar, despite being used to represent different experimental conditions:
  ○ Panel G1 of this article [1], Fig 1a of [2], and Fig 1a of [3, corrected in 4]*
  ○ Panel G5 of this article [1], and Fig 1c of [3, corrected in 4]*
  ○ Panel G7 of this article [1], and Fig 2D of [5, retracted in 6]*
- Fig 3 panel G1 of this article [1] appears similar to the following results, despite being used to represent different experimental conditions:
  ○ Fig 2a of [2]
  ○ Fig 2a of [3, corrected in 4] when rotated
  ○ Fig 7G of [7, retracted in 8]* when rotated
- Fig 4 panel G1 of this article [1] appears similar to Fig 3a of [2]
- Fig 4 panel G5 of this article [1] appears similar to Fig 4F of [9]
- Fig 5 panel G2 of this article [1] appears similar to Fig 5C of [10, retracted in 11]
- Fig 5 panel G3 of this article [1] appears to fully or partially overlap with the following results, despite being used to report different experimental conditions:
  ○ Fig 4e of [2]
  ○ Fig 4e of [3, corrected in 4]*
  ○ Fig 5F of [9]
  ○ Fig 7e of [12, retracted in 13]
  ○ Fig 8c of [14]
  ○ Fig 7G of [15, retracted in 16]
- Fig 5 panel G7 of this article [1] appears similar to Fig 9F of [7, retracted in 8]*
- Fig 6 panel G1 of this article [1] appears similar to the following results, despite being used to report different experimental conditions:
  ○ Fig 5a of [2]
  ○ Fig 8a of [12, retracted in 13]
  ○ Fig 10A of [17]
  ○ Fig 6D of [18, retracted in 19]*
- Fig 6 panel G7 of this article [1] appears similar to Fig 8A of [15, retracted in 16], despite being used to report different experimental conditions.

Some of the similar (or reused) images were used to represent controls in these articles, but different methodological details were reported for the indicated experiments in the articles’ Materials and Methods sections. Given the nature and extent of the issues, the *PLOS ONE* Editors are concerned about the reliability of data management and/or reporting for this study [1].

Following editorial communication regarding these concerns, one of the co-authors requested retraction of the article. The original data underlying this study were not provided for editorial review.

In light of the concerns affecting multiple figure panels that question the integrity of these data, the *PLOS ONE* Editors retract this article.

Some figure panels discussed above appear to report previously published material that are offered under a CC BY license, but the original articles were not attributed in [1]. For these images, the * by the citation, above, marks the oldest publication of the image of which PLOS is aware.

The Fig 4 panel G1 results report material from [2], published in 2013 by Batran et al under a CC-BY-NC-ND 3.0 license. Due to restrictions that apply to the original article’s license, this figure is excluded from the PLOS article’s [1] CC BY 4.0 license. At the time of retraction, the article [1] was republished to note this exclusion in the Fig 4 legend and the article’s copyright statement.

FAB, MMJAO, and MAA agreed with the retraction. RAB, AMA, HAH, and HMA either did not respond directly or could not be reached. FAB apologizes for the issues with the published article. MAA stands by the article’s findings.
